# Chibby suppresses aerobic glycolysis and proliferation of nasopharyngeal carcinoma via the Wnt/β-catenin-Lin28/let7-PDK1 cascade

**DOI:** 10.1186/s13046-018-0769-4

**Published:** 2018-05-15

**Authors:** Cheng-fu Cai, Guo-dong Ye, Dong-yan Shen, Wei Zhang, Mao-li Chen, Xin-xin Chen, Da-xiong Han, Yan-jun Mi, Qi-cong Luo, Wang-yu Cai, Shu-yu Yang

**Affiliations:** 1 Department of Otolaryngology-Head and Neck Surgery, The First Affiliated Hospital, Medical College, Xiamen University, Xiamen, 361003 China; 2 Teaching Hospital of Fujian Medical University, Xiamen, 361003 China; 3 The First Affiliated Hospital, Medical College, Xiamen University, Xiamen, 361003 China; 4 Xiamen Lifeint Technology Co., Ltd, Xiamen, 361026 China; 5 School of Pharmaceutical Science, Xiamen University, Xiamen, 361101 China

**Keywords:** Chibby, Warburg effect, Wnt/β-Catenin signaling, Nasopharyngeal carcinoma

## Abstract

**Background:**

Great progress has been achieved in the study of the aerobic glycolysis or the so-called Warburg effect in a variety of cancers; however, the regulation of the Warburg effect in Nasopharyngeal carcinoma (NPC) has not been completely defined.

**Methods:**

Gene expression pattern of NPC cells were used to test associations between Chibby and β-catenin expression. Chibby siRNAs and over-expression vector were transfected into NPC cells to down-regulate or up-regulate Chibby expression. Loss- and gain-of function assays were performed to investigate the role of Chibby in NPC cells. Western blot, cell proliferation, Glucose uptake, Lactate release, ATP level, and O2 consumption assays were used to determine the mechanism of Chibby regulation of underlying targets. Finally, immunohistochemistry assay of fresh NPC and nasopharyngeal normal tissue sample were used to detect the expression of Chibby, β-Catenin, and PDK1 by immunostaining.

**Results:**

We observed that Chibby, a β-catenin-associated antagonist, is down-regulated in nasopharyngeal carcinoma cell lines and inhibits Wnt/β-Catenin signaling induced Warburg effect. Mechanism study revealed that Chibby regulates aerobic glycolysis in NPC cells through pyruvate dehydrogenase kinase 1(PDK1), an important enzyme involved in glucose metabolism. Moreover, Chibby suppresses aerobic glycolysis of NPC via Wnt/β-Catenin-Lin28/let7-PDK1 cascade. Chibby and PDK1 are critical for Wnt/β-Catenin signaling induced NPC cell proliferation both in vitro and in vivo. Finally, immunostaining assay of tissue samples provides an important clinical relevance among Chibby, Wnt/β-Catenin signaling and PDK1.

**Conclusions:**

Our study reveals an association between Chibby expression and cancer aerobic glycolysis, which highlights the importance of Wnt/β-catenin pathway in regulation of energy metabolism of NPC. These results indicate that Chibby and PDK1 are the potential target for NPC treatment.

**Electronic supplementary material:**

The online version of this article (10.1186/s13046-018-0769-4) contains supplementary material, which is available to authorized users.

## Background

One of the most important hallmarks of cancer is aerobic glycolysis or the so-called Warburg effect. The Warburg effect was first described by Warburg over 90 years ago and states that cancer cells heavily rely on glycolysis for energy metabolism even under normal oxygen concentrations [1]. Consequently, unlike most normal cells, cancer cells derive a substantial amount of their energy from aerobic glycolysis, converting most incoming glucose to lactate rather than metabolizing it in the mitochondria through oxidative phosphorylation [2, 3]. Although the Warburg effect has been documented in many cancers, the underlying mechanisms driving and regulating aerobic glycolysis are not fully understood [4]. Because cancer cells adapt in various ways that distinguish cancer cells from normal cells, this is a need to know how and why cancer cells adapt to the aerobic glycolysis, which is faster in total glucose utilization but is more wasteful for energy supply. It is well known that cancer-specific metabolism is largely responsible for the growth advantage of cancer cells. Thus, uncovering the mechanisms underlying aerobic glycolysis in cancer cells could be helpful for the development of new therapeutic targets of human cancers [5].

The canonical Wnt signaling pathway plays a central role in normal development and tumorigenesis [6, 7]. The canonical Wnt pathway involves activation of the key effector molecule, β-catenin, that functions as part of a bipartite transcription factor that activates WNT-target genes by interacting with the LEF1/TCF family of transcription factors. In the absence of Wnt stimulation, β-catenin is anchored by the Axin-APC complex, subsequently phosphorylated by casein kinase Iα (CKIα) and glycogen synthase kinase-3β (GSK3β), and then targeted for ubiquitin-mediated proteasomal degradation [8]. Upon the stimulation by Wnt ligands, the Axin-APC destruction complex is inactivated through the recruitment of the intracellular signaling protein, disheveled (DVL), which prevents β-catenin degradation and allows nuclear translocation. In turn, the β-catenin–LEF1/TCF complex regulates the expression of downstream target genes involved in diverse cellular processes [9, 10].

Chibby was identified as β-catenin antagonist in a protein-protein interaction screen using the bait of C-terminal region of β-catenin in 2003 [11]. Chibby physically interacts with the C-terminal activation domain of β-catenin and represses β-catenin–mediated transcriptional activation by competing with Tcf/Lef factors for β-catenin binding [11, 12]. Moreover, Chibby facilitates β-catenin export from the nucleus in conjunction with the proteins 14–3-3 and the nuclear export receptor chromosomal region maintenance 1 (CRM1) [13, 14]. The regulatory effect of Chibby on the Wnt/β-catenin signaling pathway suggests the biological importance of Chibby as a potential tumor suppressor [11]. Several studies have shown that the expression of Chibby was down-regulated only in thyroid cancer, pediatric ependymomas and colon carcinoma cell lines [15–17]. Our previous study also indicated that the expression of Chibby is decreased in Laryngeal Squamous Cell Carcinoma (LSCC) [18]. However, the biological function of Chibby in NPC and the underlying molecular mechanism has not yet been defined. In the present study, we have demonstrated that Chibby suppresses aerobic glycolysis and the proliferation of nasopharyngeal carcinoma and that the Wnt/β-catenin-Lin28/let7-PDK1 cascade mediates this activity. Our study reveals an association between Chibby expression and aerobic glycolysis in cancer, which highlights the importance of the Wnt/β-catenin pathway in regulating energy metabolism in nasopharyngeal carcinoma.

## Methods

### Patient tissue samples

Clinical Chibby, β-Catenin, PDK1 protein levels were detected from primary human nasopharyngeal cancer or normal tissue. All samples were obtained from the First Affiliated Hospital of Xiamen University with patient consent and institutional review board approval. These samples were subsequently de-identified to protect patient confidentiality.

### Animals

Four-week-old female BALB/c nude mice were used. A total of 4 × 10^6^ cells were injected subcutaneously into the dorsal thighs of mice. Tumor growth was monitored regularly for 6 weeks, then the tumor volume was calculated every week. All mice were kept under specific pathogen-free conditions at Xiamen University Laboratory Animal Center (Xiamen University, China) in accordance with institutional guidelines. This study was approved by the local Ethical Committee of Xiamen University.

### Statistical analysis

Date were analyzed using GraphPad Prism software. Data are presented as the means ± standard error. The Student’s t-test (two-tailed), Fisher’s exact test, and Pearson’s r were used to compare data and to calculate their probability value (*p*). *p* < 0.05 was considered statistically significant.

### Other procedures

Protocols for other procedures are described in the Additional file 1.

## Results

### Chibby has an inverse expression pattern with β-catenin and inhibits proliferation in nasopharyngeal carcinoma cell lines

**Fig. 1:**
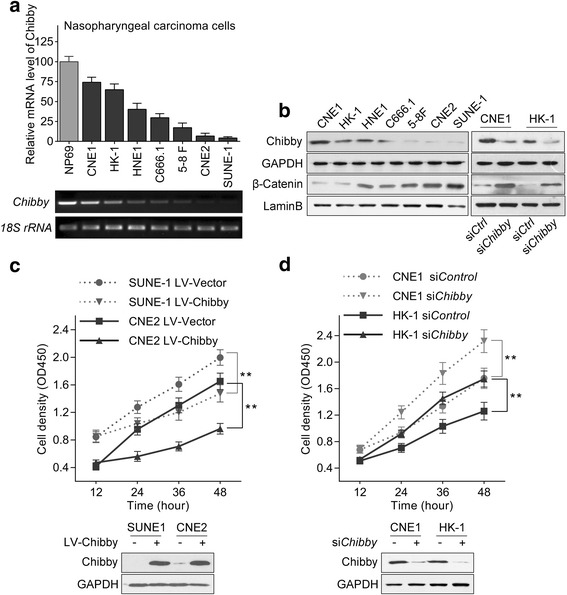
Expression pattern of Chibby and its effects on cell proliferation in NPC cells. **a** Quantitative and semi-quantitative polymerase chain reaction analysis of *Chibby* in a variety of cell lines including normal and NPC cells*.* **b** Western blotting analysis of Chibby and β-catenin from cell lysates or nuclear extracts of NPC cell lines. **c** Overexpression of Chibby suppressed cell proliferation (*n* = 3) in SUNE-1 and CNE2 cells. **d** Knockdown of Chibby enhanced cell proliferation (*n* = 3) in CNE1 and HK-1 cells. Data were expressed as the mean ± SD. **, *p* < 0.01

To determine whether Chibby is involved in the carcinogenesis of nasopharyngeal carcinoma, we analyzed the expression of Chibby in diverse nasopharyngeal carcinoma cell lines. Compared with the NP69 immortalized normal nasopharyngeal cell line, Chibby expression was significantly down-regulated in CNE1, HK-1, HNE1, C666.1, 5-BF, CNE-2 and SUNE-1 cell lines (Fig. 1a). Moreover, the inverse protein expression pattern between Chibby and nuclear β-catenin was observed in these NPC cell lines, and the expression of β-catenin was increased with the knockdown of Chibby (Fig. 1b). To further determine the physiological relevance of Chibby in NPC cells, we overexpressed Chibby in SUNE-1 and CNE2 cells and found that overexpression of Chibby showed significantly impaired proliferation of cells (Fig. 1c). In contrast, knockdown of Chibby in CNE1 and HK-1 cells resulted in significantly increased cell proliferation (Fig. 1d). Taken together, these data demonstrate that the expression pattern of Chibby is inverse with β-catenin, and Chibby inhibits cell proliferation in nasopharyngeal carcinoma cell lines.

### Chibby inhibits the Warburg effect in NPC cells

**Fig. 2:**
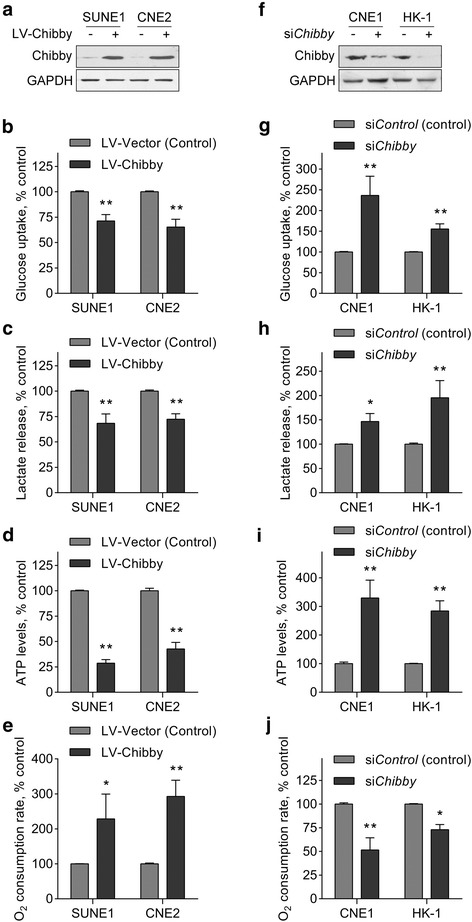
The effects of Chibby overexpression or knockdown on Warburg effect in NPC cells. **a** Western blotting analysis of Chibby in overexpressed SUNE-1 and CNE2 cells. **b** Cellular glucose uptake, **c** Lactate release, **d** ATP levels, **e** O_2_ consumption rates were measured in Chibby-overexpressed SUNE-1 and CNE2 cells. **f** Western blotting analysis of Chibby in knocked down CNE1 and HK-1 cells. **g** Cellular glucose uptake, **h** Lactate release, **i** ATP levels, **j** O_2_ consumption rates were measured in Chibby-knockdown CNE1 and HK-1 cells. Data were expressed as the mean ± SD, *n* = 6 for each group. *, *p* < 0.05; **, *p* < 0.01

Aberrant glucose metabolism can occur in NPC cells [19]. In the above experiments, we found that the metabolism ability of the NPC cells after Chibby knockdown was changed. We hypothesized that Chibby could regulate aerobic glycolysis, one of the hallmarks of cancer, to facilitate NPC cell proliferation. To verify this hypothesis, we overexpressed Chibby in SUNE-1 and CNE2 cells (Fig. 2a) and checked the metabolic parameters. The results indicated that the cellular glucose uptake and lactate production in a culture medium were significantly reduced (Fig. 2b, c). Moreover, overexpression of Chibby led to decreased cellular ATP levels and increased cellular O2 consumption rates (Fig. 2d, e). By contrast, knockdown of Chibby in CNE1 and HK-1 cells resulted in reversed effects on the above metabolic parameters (Fig. 2f-i). Together, these results suggest that Chibby inhibits aerobic glycolysis, or the Warburg effect, in NPC cells.

### Chibby regulates aerobic glycolysis in NPC cells through PDK1

**Fig. 3:**
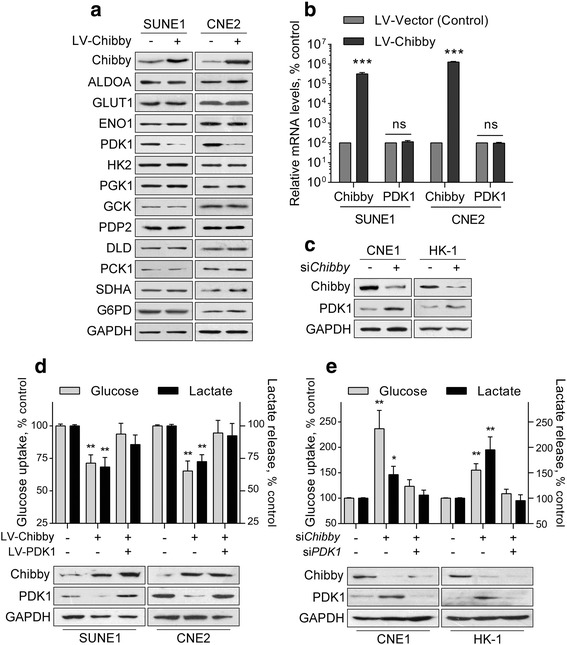
PDK1 mediates Chibby-regulated aerobic glycolysis. **a** Western blotting analysis of metabolic enzymes in Chibby-overexpressed SUNE-1 and CNE2 cells. **b** mRNA levels of PDK1 were determined by quantitative polymerase chain reaction in Chibby-overexpressed SUNE-1 and CNE2 cells. **c** protein levels of PDK1 were determined by western blot in Chibby-knockdown CNE1 and HK-1 cells. **d** Cellular glucose uptake and lactate release levels were measured in Chibby-overexpressed SUNE-1 and CNE2 cells after PDK1 overexpression. **e** Cellular glucose uptake and lactate release levels were measured in Chibby-knockdown CNE1 and HK-1 cells following knockdown of PDK1. Data were expressed as the mean ± SD, *n* = 3 for each group. *, *p* < 0.05; **, *p* < 0.01; ***, *p* < 0.001; ns, not significant

To determine the downstream molecular events by which Chibby regulates aerobic glycolysis, we examined the protein expression of all important enzymes involved in glucose metabolism in SUNE-1 and CNE2 cells overexpressing Chibby. While all other enzymes were not significantly changed, overexpression of Chibby resulted in an obvious decrease in PDK1 protein expression (Fig. 3a). However, Chibby did not affect PDK1 mRNA levels (Fig. 3b), suggesting that Chibby regulates PDK1 by a post-transcriptional mechanism. Consistently, knockdown of Chibby in CNE1 and HK-1 cells significantly increased PDK1 protein levels (Fig. 3c). Since PDK1 is a critical enzyme regulating glycolytic metabolism in cancer cells, we next sought to determine whether PDK1 is involved in Chibby-mediated changes in glycolysis. Our results showed that when PDK1 was overexpressed or knocked down, Chibby-regulated glucose uptake and lactate production were almost completely compromised (Fig. 3d, e), indicating that PDK1 mediates Chibby-regulated aerobic glycolysis.

### PDK1 is positively regulated by Wnt/β-catenin signaling via Lin28/Let-7g

**Fig. 4:**
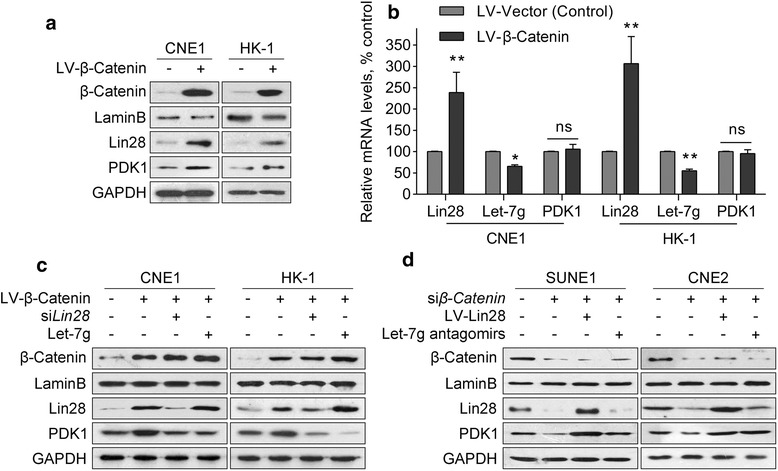
PDK1 is positively regulated by Wnt/β-catenin signaling via Lin28/Let7. **a** Western blotting analysis of Lin28 and PDK1 in β-catenin-overexpressed CNE1 and HK-1 cells. **b** mRNA levels of Lin28, let-7 g, and PDK1 were determined by quantitative polymerase chain reaction in β-catenin-overexpressed CNE1 and HK-1 cells. **c** Western blotting analysis of Lin28 and PDK1 in β-catenin-overexpressed CNE1 and HK-1 cells after Lin28 knockdown or Let-7 g overexpression. **d** Western blotting analysis of Lin28 and PDK1 in β-catenin-knockdown SUNE-1 and CNE2 cells after Lin28 overexpression or Let-7 g antagomir treatment. Data were expressed as the mean ± SD, *n* = 3 for each group. *, *p* < 0.05; **, *p* < 0.01; ns, not significant

Previous work has demonstrated that PDK1 is post-transcriptionally regulated by Lin28/Let-7g in hepatocellular carcinoma (HCC) cells [20]. Moreover, Wnt/β-catenin signaling represses Let-7 g microRNA expression through transactivation of Lin28 to augment breast cancer stem cell expansion [21]. However, the link between Wnt/β-catenin and PDK1 in these cancer cells, as well as NPC cells is still unknown. We deduced that Wnt/β-catenin signaling positively regulates PDK1 protein expression via Lin28/Let-7g in NPC cells. When β-catenin was overexpressed in CNE1 cells, Lin28 protein expression significantly increased (Fig. 4a), whereas Let-7 g expression significantly decreased (Fig. 4b). PDK1 expression also significantly increased (Fig. 4a). However, when Lin28 was knocked down or Let-7 g mimics were overexpressed, the β-catenin-induced PDK1 expression was abolished (Fig. 4c). Conversely, overexpression of Lin28 or a Let-7 g antagomir almost completely compromised the β-catenin knockdown-induced PDK1 reduction (Fig. 4d). Thus, the above results demonstrated that PDK1 is positively regulated by Wnt/β-catenin signaling via Lin28/Let-7g.

### Chibby inhibits Wnt/β-catenin signaling-induced PDK1 expression and aerobic glycolysis in NPC cells

**Fig. 5:**
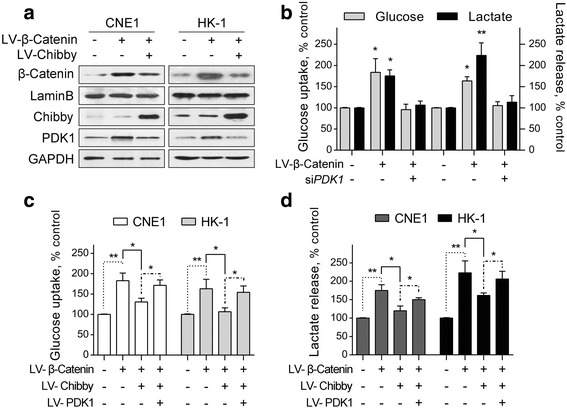
Chibby inhibits Wnt/β-catenin signaling-induced PDK1 expression and aerobic glycolysis in NPC cells. **a** Western blotting analysis of PDK1 in β-catenin-overexpressed CNE1 and HK-1 cells after Chibby overexpression. **b** Cellular glucose uptake and lactate release levels were measured in β-catenin-overexpressed CNE1 and HK-1 cells following knockdown of PDK1. **c** Cellular glucose uptake, **d** Lactate levels were measured in CNE1 and HK-1 cells following overexpression of β-catenin, Chibby, PDK1, alone or in combination. Data were expressed as the mean ± SD, *n* = 3 for each group. *, *p* < 0.05; **, *p* < 0.001

To examine whether Chibby inhibits Wnt/β-catenin signaling-induced PDK1 expression, we overexpressed β-catenin in CNE1 and HK-1 cells. As expected, PDK1 expression levels were significantly increased, whereas simultaneous overexpression of Chibby was found to almost completely compromise Wnt/β-catenin activity (Fig. 5a). We next monitored the regulatory features of these three factors in glycolytic metabolism. We found that when PDK1 was knocked down the effect of Wnt/β-catenin signaling on glucose uptake and lactate production was markedly attenuated (Fig. 5b). Similar results were observed when we overexpressed Chibby in β-catenin-overexpressed cells. However, when PDK1 was re-expressed, the cell metabolism phenotype was significantly rescued (Fig. 5c, d), establishing PDK1 as a functional downstream target of Wnt/β-catenin signaling in regulation of glycolytic metabolism. These results also suggest that Chibby inhibits Wnt/β-catenin signaling-induced aerobic glycolysis in NPC cells.

### Chibby and PDK1 are critical for Wnt/β-catenin signaling-induced NPC cell proliferation

**Fig. 6:**
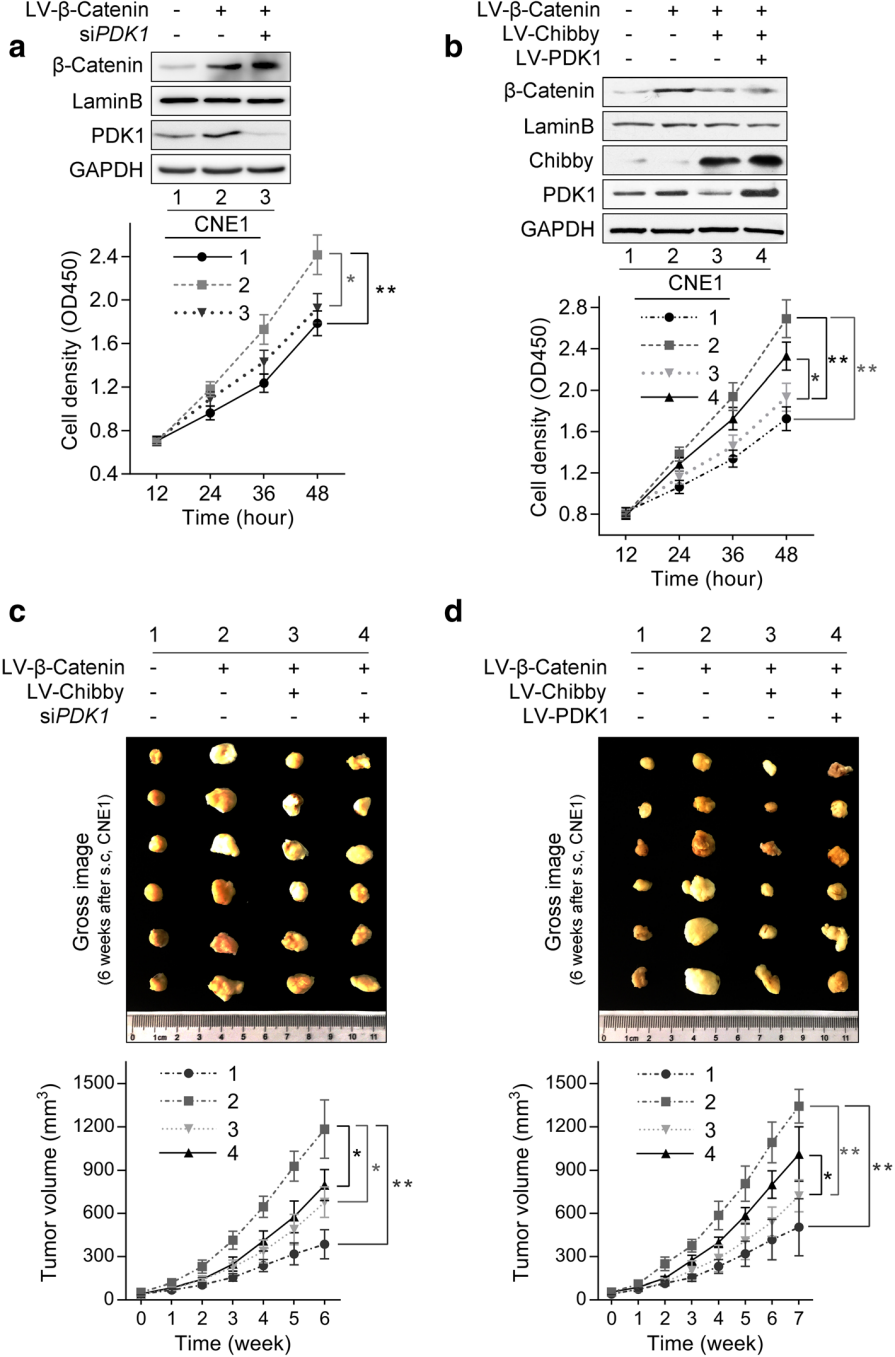
The effects of Chibby and PDK1 on Wnt/β-catenin signaling-induced NPC cell proliferation. **a** Knockdown of PDK1 diminished the promoting effect of β-catenin on cell proliferation in CNE1 cells. **b** Cell proliferation rates were measured in CNE1 cells following overexpression of β-catenin, Chibby, PDK1, alone or in combination. **c**, **d** Representative photograph and tumor growth curve of CNE1 xenografts in nude mice. 4 × 10^6^ cells of CNE1 were injected subcutaneously into mice. Tumor growth was monitored regularly for 7 weeks, then the tumor volume was calculated every week. Data were expressed as the mean ± SD, *n* = 6 for each group. *, *p* < 0.05; **, *p* < 0.01

Since our results have clearly demonstrated that Chibby inhibits Wnt/β-catenin signaling-induced PDK1 expression and aerobic glycolysis in NPC cells, we further explored whether Chibby and PDK1 are critical for Wnt/β-catenin signaling-induced NPC cell proliferation. First, we knocked down PDK1 in β-catenin-overexpressed CNE1 cells. Cell growth analysis revealed that knockdown of PDK1 diminished the promoting effect of β-catenin on cell proliferation (Fig. 6a). Similar results were achieved when we overexpressed Chibby in β-catenin overexpressing cells; however, when PDK1 was re-expressed, the cell proliferation phenotype was significantly rescued (Fig. 6b). Next, xenograft experiments in nude mice were conducted. Compared with the control group, the results demonstrated significantly enhanced tumor size in β-catenin-overexpressed NPC cells. However, when PDK1 was knocked down or Chibby was overexpressed, β-catenin-enhanced tumor growth was obviously restrained (Fig. 6c). The re-expression of PDK1 also significantly rescued the tumor growth phenotype caused by overexpression of Chibby in β-catenin-overexpressed cells (Fig. 6d). These results demonstrate that Chibby and PDK1 are critical for Wnt/β-catenin signaling-induced NPC cell proliferation both in vitro and in vivo.

### Clinical relevance of Chibby, Wnt/β-catenin signaling and PDK1

**Fig. 7:**
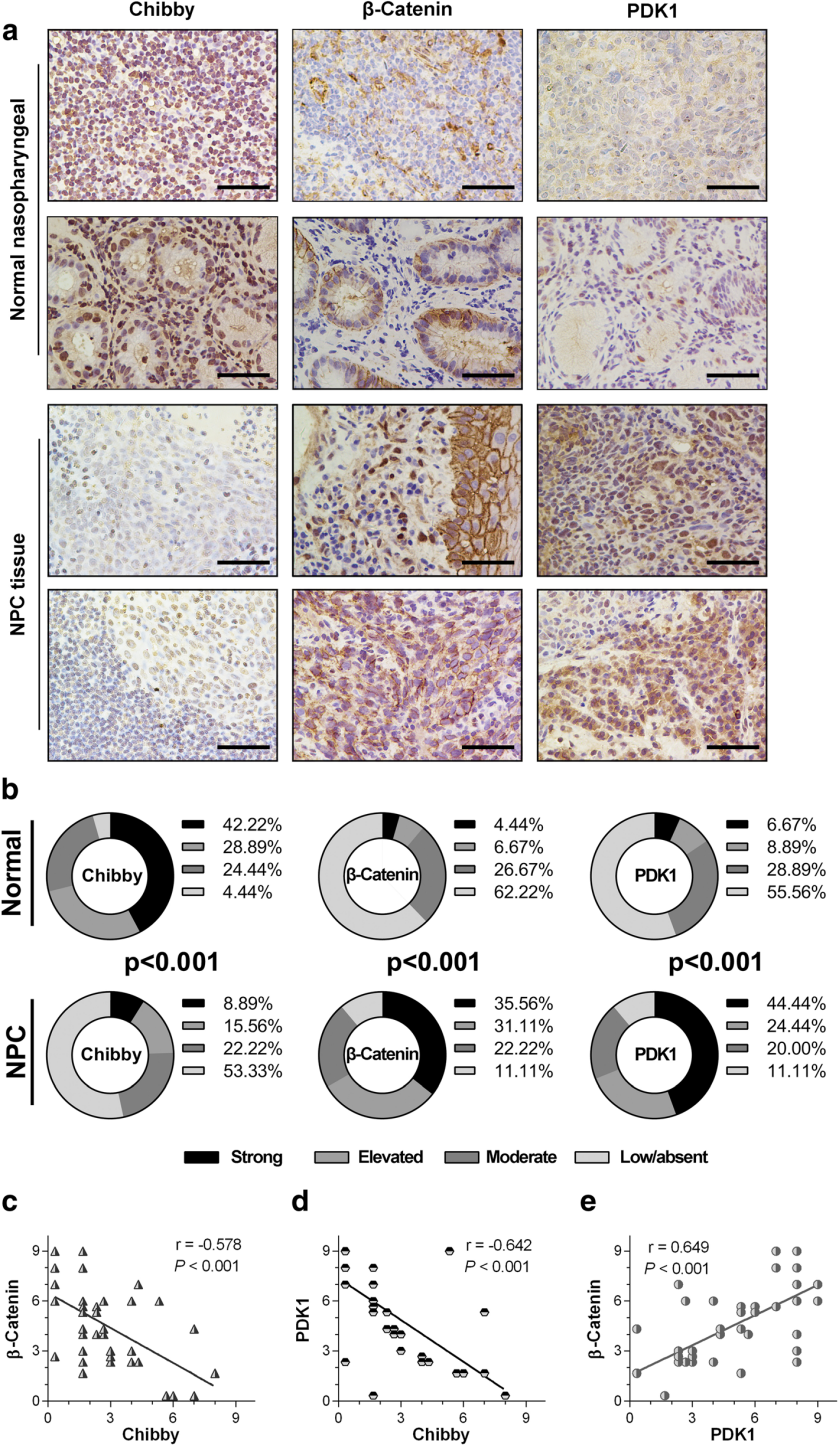
Clinical relevance of Chibby, Wnt/β-catenin signaling and PDK1. **a** Representative immunostaining of Chibby, β-catenin and PDK1 in normal and NPC tissue samples. Scale bar: 100 μm. **b** Relative frequency of the immunoreactive scores for Chibby, β-catenin and PDK1 staining on 45 NPC tissues and 45 normal nasopharyngeal tissues. Fisher’s exact test was used for categorical variables (*p* < 0.001). **c** The correlation between Chibby and β-catenin protein levels. **d** The correlation between Chibby and PDK1 protein levels. **e** The correlation between β-catenin and PDK1 protein levels. Pearson’s correlation test was used for **c** to **e** (r and *p* values are shown in the graphs of **c** to **e**)

To investigate whether Chibby is associated with Wnt/β-catenin signaling and PDK1 in NPC patients, 45 fresh normal tissue samples and 45 NPC tissue samples were used to detect the expression of Chibby, β-catenin, and PDK1 by immunostaining. As indicated by the representative samples in Fig. 7a, overall expression levels of the Chibby protein were significantly lower in NPC than normal tissues, whereas the β-catenin and PDK1 protein expression levels were significantly higher in NPC tissues. The quantitative summary of all of the samples is shown in Fig. 7b. Correlation analyses revealed inverse correlations between Chibby and nuclear β-catenin (*r* = − 0.578, *p* < 0.001, Fig. 7c) or between Chibby and PDK1 levels (*r* = − 0.642, *p* < 0.001, Fig. 7d) and a strong correlation between nuclear β-catenin and PDK1 (*r* = 0.649, *p* < 0.001, Fig. 7e). Taken together, the above results provide the clinical relevance of Chibby, Wnt/β-catenin signaling and PDK1.

## Discussion

Nasopharyngeal carcinoma (NPC) is one of the most common malignant tumors and is reported as an endemic disease with high prevalence in Southeast Asia, particularly in South China [22, 23]. The etiology and pathogenesis of NPC have not yet been completely defined. Emerging studies have suggested that environmental factors, genetic susceptibility, and Epstein-Barr virus may play crucial roles in its carcinogenesis. Although the 5-year survival rate of NPC has been greatly improved through comprehensive treatments such as radiotherapy and chemotherapy [24], long-term prognosis remains unsatisfactory. The approaches that change or modify some important genes or their expression have become a research hotspot in the biological treatment of NPC. Therefore, there is an urgent need to further explore the molecular mechanism during carcinogenesis of NPC. Many signaling pathways have been reported to be involved in this process. However, there is very little knowledge regarding Wnt/β-catenin signaling cascade genes in NPC [25]. Numerous studies have revealed the role of Wnt/β-catenin signaling in the carcinogenesis of many cancers; however, the regulation of this signaling process during carcinogenesis has not been completely defined. Moreover, since somatic mutations of Wnt/β-catenin signaling components are rare in NPC, regulators of Wnt/β-catenin signaling components primarily control the Wnt/β-catenin output level. Accumulating evidence has demonstrated that the inhibition of Wnt/β-catenin by ZNRF3 [26], YPEL3 [27], SFRP1 [28], Wnt-C59 [29], SOX1 [30] and WIF-1 [31] in NPC cells was significantly compromised, resulting in elevated Wnt/β-catenin output levels. Chibby is an interaction partner and negative regulator of β-catenin; however, its role in NPC has not been elucidated. To the best of our knowledge, this report is the first to link Chibby to NPC.

Wnt/β-catenin signaling has been implicated in the mediation of cancer cell metabolism via multiple mechanisms [32]. Specifically, it was reported that PDK1 served as a direct downstream target gene of Wnt/β-catenin signaling in colon cancer cells and mediated aerobic glycolysis [33]. And PDK1 would down-regulate pyruvate dehydrogenase (PDH) to shutting down pyruvate entry into the tricarboxylic acid cycle (TCA) [34]. However, in the present study we did not observe changes in PDK1 mRNA levels upon Wnt/β-catenin activation in NPC cells. Instead, PDK1 was post-transcriptionally regulated by Wnt/β-catenin signaling via the Lin28-Let-7 pathway in NPC cells, which reflects the tissue specificity and cancer-type dependence. Moreover, we noticed that, compared to PDK1 mRNA levels, blocking Wnt/β-catenin activity in colon cancer cells resulted in a further reduction in PDK1 protein levels [33], which suggests that post-transcriptional regulation of PDK1 by Wnt/β-catenin signaling at least partially contributes to the metabolism of colon cancer cells. Previous studies also identified other mechanisms downstream of Wnt/β-catenin signaling to regulate aerobic glycolysis. For example, the well-known Wnt/β-catenin target gene c-Myc plays an important role in cancer metabolism, driving both aerobic glycolysis and glutaminolysis [35–37]. Moreover, c-Myc has been shown to enhance HIF-1a-mediated regulation of PDK1 [38]. However, we found that c-Myc is not required in our system to mediate changes in metabolism as its levels are not altered in NPC cells upon Wnt/β-catenin activation (data not shown), which also suggests a context dependence.

Given the strong evidence for regulation of PDK1 protein expression by Chibby through Wnt/β-catenin signaling, we asked whether Chibby, Wnt/β-catenin signaling and PDK1 were correlated in primary human NPC specimens. We used 45 pairs of fresh NPC samples with normal tissues to detect the expression of Chibby, nuclear β-catenin, and PDK1 by immunostaining. Indeed, we observed a strong inverse correlation between Chibby and nuclear β-catenin or PDK1 levels and a strong correlation between nuclear β-catenin and PDK1, which supports the results observed in in vitro experiments.

## Conclusions

Taken together, our findings identified Chibby as a negative regulator of proliferation that suppresses NPC aerobic glycolysis via the inhibition of Wnt/β-catenin signaling in vitro and in vivo. The modulation of this molecular process may be a method of inhibiting NPC cell growth by restoring Chibby expression to interfere with cell metabolism. In particular, the PDK1 may become a new target for further inhibitor design to interfere with Wnt/β-catenin dependent NPC progression.

## Additional file

Additional file 1: Supplementary Methods. (DOCX 19 kb)

## Abbreviations

ALDOA: Aldolase A
ATP: Adenosine triphosphate
CBY: Chibby
DLD: Dihydrolipoamide dehydrogenase
ENO1: Enolase-1
G6PD: Glucose-6-phosphate dehydrogenase
GCK: Glucokinase
GLUT1: Glucose transporter 1
HK2: Hexokinase 2
NPC: Nasopharyngeal carcinoma
PCK1: Phosphoenolpyruvate carboxykinase 1
PDK1: Pyruvate dehydrogenase kinase 1
PDP2: Pyruvate dehydrogenase phosphatase catalytic subunit 2
PGK1: Phosphoglycerate kinase 1
SDHA: Succinate dehydrogenase complex, subunit A
