# Supplementary material for: Chibby suppresses aerobic glycolysis and proliferation of nasopharyngeal carcinoma via the Wnt/β-catenin-Lin28/let7-PDK1 cascade
Source: J Exp Clin Cancer Res. 2018 May 15;37:104. doi: 10.1186/s13046-018-0769-4 (PMC5952826; doi:10.1186/s13046-018-0769-4)
Supplement: Supplementary file 1 — Supplementary Methods. (DOCX 19 kb) [file 13046_2018_769_MOESM1_ESM.docx]

**Chibby suppresses aerobic glycolysis and proliferation of** **nasopharyngeal carcinoma via the Wnt/β-catenin-Lin28/let7-PDK1 cascade**

Cheng-fu Cai, Guo-dong Ye, Dong-yan Shen, Wei Zhang, Mao-li Chen, Xin-xin Chen, Da-xiong Han, Yan-jun Mi, Qi-cong Luo, Wang-yu Cai, Shu-yu Yang

**Supplementary Methods**

**Chemicals and antibodies**

Cell Counting Kit-8 (CCK-8, Cat#CK04) was purchased from Dojindo (Kumamoto, Japan). The anti-sense oligonucleotides (siRNAs) pools for human Chibby, PDK1, β-Catenin were purchased from GenePharma (Shanghai, China). The Let-7g mimics and antagomirs were purchased from RiboBio (Guangzhou, China). Antibodies of Chibby (Cat#SAB4300812) was purchased from Sigma-Aldrich (St. Louis, MO, USA), and β-Catenin (Cat#ab32572), PDK1 (Cat#ab52893) were purchased from Abcam (Cambridge, MA, USA). And the following antibodies were used, Glycolysis Antibody Sampler Kit #12866 and #8337 (Cell Signaling Technology, Cat#12866, Cat#8337), anti-GLUT1, anti-PGK1, anti-GCK, anti-PDP2, anti-DLD, anti-PCK1, anti-SDHA, anti-G6PD (Abcam, Cat#ab115730, ab38007, ab184169, ab99170, ab133551, ab28455, ab14715, ab210702), anti-GAPDH and anti-Lamin B (Cell Signaling Technology, Cat#8884, Cat#13435)

**Cell culture**

Human embryonic kidney (HEK 293T, HEK 293) were obtained from the American Type Culture Collection, and human nasopharyngeal cancer cell lines were gift from Dr. Yuan-ji Xu from Fujian Medical University. Cells were cultured in Dulbecco’s modified Eagle’s medium (DMEM) supplemented with 10% fetal calf serum (Gibco), 100 units/ml penicillin, and 100 mg/ml streptomycin. All cell lines were grown at 37°C with 5% carbon dioxide.

**Plasmid construction and Lentivirus**

For overexpression of Chibby, PDK1, and β-Catenin, the cDNAs were cloned under the control of the EF-1α promoter in the lentiviral vector pLV-CS2.0. Lentivirus vectors were generated by co-transfecting pLV-CS2.0 carrying the expression cassette with helper plasmids pVSV-G and pHR into HEK293T cells using Lipofectamine 2000 (Invitrogen, Waltham, MA). The viral supernatant was collected 48 h after transfection. Cells at 50% to 70% confluence were infected with viral supernatants containing 10 μg/mL Polybrene for 24 h, after which fresh medium was added to the infected cells.

**Cell proliferation assay**

Cell proliferation was analyzed by the CCK-8 (Cell Counting Kit-8, Dojindo) assay following the manufacturer’s instructions. The absorbance was measured at 450 nm using a microplate reader.

**Real-time quantitative PCR (qPCR)**

Total RNA (2 μg) from cells was used for first-strand cDNA synthesis (Invitrogen). Platinum SYBR Green qPCR SuperMix (Invitrogen) was used for the qPCR reaction, and the expression levels were quantified using the -ΔΔCt method. Primer sequences for Chibby were 5’- CAGGGCAAAGCCTGAAGTTT -3’ (forward) and 5’- CAGCAGTGGACTCTGAAAGC - 3’ (reverse), for PDK1 were 5’- TCAGGACACCATCCGTTCAA-3’ (forward) and 5’- CATCTTGCAGGCCATACAGC-3’ (reverse), for Lin28 were 5’- CAAAAGGAAAGAGCATGCAGAA -3’ (forward) and 5’-ATGATCTAGACCTCCACAGTTGTAGC-3’ (reverse), for Let-7g were 5’-GTCGTATCCAGTGCAGGGTCCGAGGTATTCGCACTGGATACGACAACTGT-3’ (reverse transcription) and 5’-GCCGCTGAGGTAGTAGTTTGT-3’ (qPCR).

**Western blotting**

Western blotting was performed according to standard procedures. Protein concentration of cell lysate was measured using the BCA protein assay kit (Cat# 23227, Thermo Fisher Scientific Inc., Waltham, MA, U.S). Equal amount of proteins was loaded and separated by SDS–polyacrylamide gel electrophoresis. anti-GAPDH and anti-Lamin B were served as loading control.

**Glucose uptake, Lactate release, ATP level, and O2 consumption assay**

Intracellular glucose was measured using cell lysates with glucose assay kit (BioVision), the extracellular lactate was measured using the cell culture medium with lactate assay kit (BioVision), the ATP levels were measured using an ATP assay kit (Promega), and the oxygen consumption was examined using Oxygen Consumption Rate Assay Kit (Cayman Chemical) according to the manufacturer’s instruction. The values were normalized to the protein concentration.

**Immunohistochemistry assay**

All of the fresh tissues were fixed in 10% neutral buffered formalin, stored in 70% ethanol, paraffin embedded and sectioned. After dewaxing and rehydration, antigen retrieval was performed by boiling in citrate buffer (pH 6.0) for 25 min. The sections were then pre-treated with peroxidase blocking buffer for 20 min at room temperature. After treatment with blocking buffer (5% normal goat serum in PBS) for 1 hour at room temperature, sections were incubated with the primary antibody in blocking buffer. The secondary antibody reagents were from a DAB kit.
